# Neutrophil extracellular traps and neutrophil-derived mediators as possible biomarkers in bronchial asthma

**DOI:** 10.1007/s10238-021-00750-8

**Published:** 2021-08-03

**Authors:** Gilda Varricchi, Luca Modestino, Remo Poto, Leonardo Cristinziano, Luca Gentile, Loredana Postiglione, Giuseppe Spadaro, Maria Rosaria Galdiero

**Affiliations:** 1 Department of Translational Medical Sciences, University of Naples Federico II, 80131 Naples, Italy; 2 Center for Basic and Clinical Immunology Research (CISI), University of Naples Federico II, 80131 Naples, Italy; 3 World Allergy Organization (WAO) Center of Excellence, 80131 Naples, Italy; 4 Institute of Experimental Endocrinology and Oncology (IEOS), National Research Council, 80131 Naples, Italy; 5 Department of Molecular Medicine and Medical Biotechnologies, University of Naples Federico II, 80131 Naples, Italy

**Keywords:** Asthma, Myeloperoxidase, Neutrophil extracellular traps, ROS

## Abstract

Neutrophils (PMNs) contain and release a powerful arsenal of mediators, including several granular enzymes, reactive oxygen species (ROS) and neutrophil extracellular traps (NETs). Although airway neutrophilia is associated with severity, poor response to glucocorticoids and exacerbations, the pathophysiological role of neutrophils in asthma remains poorly understood. Twenty-four patients with asthma and 22 healthy controls (HCs) were prospectively recruited. Highly purified peripheral blood neutrophils (> 99%) were evaluated for ROS production and activation status upon stimulation with lipopolysaccharide (LPS), N-formylmethionyl-leucyl-phenylalanine (fMLP) and phorbol 12-myristate 13-acetate (PMA). Plasma levels of myeloperoxidase (MPO), CXCL8, matrix metalloproteinase-9 (MMP-9), granulocyte–monocyte colony-stimulating factor (GM-CSF) and vascular endothelial growth factor (VEGF-A) were measured by ELISA. Plasma concentrations of citrullinated histone H3 (CitH3) and circulating free DNA (dsDNA) were evaluated as NET biomarkers. Activated PMNs from asthmatics displayed reduced ROS production and activation status compared to HCs. Plasma levels of MPO, MMP-9 and CXCL8 were increased in asthmatics compared to HCs. CitH3 and dsDNA plasma levels were increased in asthmatics compared to controls and the CitH3 concentrations were inversely correlated to the % decrease in FEV_1_/FVC in asthmatics. These findings indicate that neutrophils and their mediators could have an active role in asthma pathophysiology.

**Supplementary Information:**

The online version contains supplementary material available at 10.1007/s10238-021-00750-8.

## Introduction

Bronchial asthma is a heterogeneous chronic inflammatory disease with a broad spectrum of severity [1]. More than 300 million people globally suffer from asthma with up to 10% of all patients having severe asthma [2, 3]. Asthma heterogeneity mirrors the underlying molecular mechanisms and contributes to the variable presentation of the disease [4]. Therefore, asthma is not a single disease and several endotypes/phenotypes of asthma have currently been defined [5, 6]. According to the type of inflammation, asthma has been divided into T (type) 2-high and T2-low [7]. T2-high asthma is the most frequent endo/phenotype and is canonically marked by activation of type 2 helper (Th2) cells, type 2 innate lymphoid cells (ILC2s), mast cells, basophils, eosinophils and the production of type 2 cytokines (e.g., IL-3, IL-4, IL-5 and IL-13) [8–10]. Besides T_H_2 cells, also T follicular helper (T_FH_) cells, a specialized subset of CXCR5^+^ CD4^+^ T cells, are a major source of IL-4 and IL-21 [11], thus closely regulating IgE isotype switching during asthma in both humans and mice [12]. T2-high asthma biomarkers are represented by exhaled nitric oxide (FeNO), serum IgE, blood and sputum eosinophils [13]. In T2-low asthma, respiratory viruses, bacteria, fungi and cigarette smoke can induce the release of epithelium-derived alarmin [i.e., IL-33, thymic stromal lymphopoietin (TSLP) and IL-25], which activate a variety of cells of the innate and adaptive immune system [8, 14]. T2-low asthma is characterized by neutrophilic or paucigranulocytic inflammation and the activation of Th1 and/or Th17 cells, ILC1s, ILC3s and the release of specific cytokines (e.g., CXCL8, IL-17) [9, 15]. Specific biomarkers phenotyping T2-low asthma are currently lacking and represent unmet needs. Therefore, the T2-low endotype is a diagnosis of exclusion defined by the absence of T2 biomarkers [16]. Both neutrophils and CXCL8 have been demonstrated in sputum and blood of severe asthma patients [17, 18]. CXCL8 is overexpressed in airway secretions of severe asthma patients, and neutrophil airway infiltration is found in severe asthma [19].

Neutrophils (or polymorphonuclear leukocytes: PMNs) are the most abundant circulating leukocytes in humans [20] and are an important component of the host response against pathogens [21–23]. They represent the first line of defense of the innate immune system against infectious agents [24]. During infections, neutrophils migrate from peripheral blood to tissues in response to chemotactic stimuli (e.g., formylated peptides, CXCL8, lipopolysaccharide) released during the inflammatory response [25–27]. PMNs rapidly bind and kill microbes through the release of a potent antimicrobial arsenal, including several granular enzymes [myeloperoxidase (MPO), matrix metalloproteinases (MMPs)] and reactive oxygen species (ROS) [28, 29]. A variety of immunologic and non-immunologic stimuli, including several bacteria [25], fungi [30] and viruses [31, 32] activate human neutrophils to release neutrophilic extracellular traps (NETs), composed by a DNA scaffold with associated granule proteins in a process sometimes called NETosis [33]. NETs bind and kill bacteria [25]. Beyond infectious diseases [25, 34–36], NETs play a role in autoimmune disorders [37, 38] and cancer [24, 39–41]. There is preliminary evidence that NETs can contribute to some aspects of allergic disorders through different mechanisms [42–47].

The pathophysiological role of neutrophils in asthma has recently begun to emerge [48]. Circulating PMNs from aspirin-exacerbated asthma patients displayed increased ROS production, CD11b expression and CXCL8 and MMP-9 release compared to aspirin-tolerant asthma patients [49]. In patients with allergic asthma, allergen provocation caused a shift in the neutrophil subsets from CD16^high^ CD62L^high^ to CD16^high^ CD62L^low^ [50]. NETs were found in bronchial biopsies of asthmatic patients [47]. In addition, NETs promoted CXCL8 production from human airway epithelial cells [43]. Activated neutrophils release ROS, which contributes to the pathogenesis of asthma by inducing inflammatory injury in airway epithelial cells [51, 52].

In this study, we evaluated the production of ROS from highly purified peripheral blood neutrophils from asthma patients of different severity compared to healthy controls. In addition, we compared the ex vivo activation status of neutrophils, the circulating levels of several neutrophil-derived mediators and of NET-derived biomarkers in healthy donors and asthma patients.

## Materials and methods

### Patients and methods

We enrolled 24 asthma patients (17 females, mean age 50.71 ± 15 years and 22 healthy matched donors (17 females, mean age 49.1 ± 15 years) from April 2020 to May 2021 at the Division of Clinical Immunology, Allergy and Immunodeficiency of the Department of Translational Medical Sciences, University of Naples Federico II. The study was conducted in accordance with Good Clinical Practice (GCP) guidelines and adhered to the Declaration of Helsinki II. Written informed consent was obtained from all participants. Patients were eligible for enrollment in the study if they were aged 18–70 years and had a history of allergist-diagnosed asthma. Key exclusion criteria were autoimmune diseases, infections, malignancies, a patient-reported smoking history or the onset of respiratory symptoms after the age of 40 years in current or previous smokers with a smoking history of at least 10 pack-years. None of the asthmatic patients has been or was treated with allergen-specific immunotherapy [53] or monoclonal antibodies anti-IgE, anti-IL-5/IL-5Rα or anti-IL-4Rα [4, 54, 55]. The majority of patients (20/24) were treated with daily low-dose of inhaled glucocorticoids (ICS) therapy [fluticasone propionate (FP), 100–200 μg or equivalent] plus two additional controllers (e.g., a long-acting β_2_-agonist and/or leukotriene-receptor antagonist); 4/24 of patients were treated with daily medium-dose of ICS (FP, 250–500 μg or equivalent); 3/24 of patients were on oral glucocorticoid therapy (mean prednisone intake 14.2 mg/die). Baseline characteristics of the patients and healthy volunteers included in the study are shown in Tables 1 and 2. Peripheral blood samples were collected at the Center for Basic and Clinical Immunology Research (CISI), University of Naples Federico II and were immediately processed. Plasma samples were also obtained (+ 4 °C, 400 g, 20 min) and stored (− 80 °C) until used. The following parameters were evaluated: the on-treatment forced expiratory volume in 1 s (FEV_1_, in liters); the fraction of exhaled nitric oxide (F_eno_, in parts per billion: ppb); the score on the Asthma Control Test (ACT; the mean score of five questions that assess asthma symptoms, use of rescue medications and the effect of asthma on daily functioning during the previous 4 weeks) [56]. Spirometry (Quark PTF, COSMED, Pavona di Albano, Italy) (before and after salbutamol 400 mcg) was performed in accordance with the American Thoracic Society/European Respiratory Society (ATS/ERS) guidelines [57]. FEV_1_, Forced Vital Capacity (FVC), FEV_1_/FVC were measured and the best of three forced maneuvers was recorded. Results were expressed both as absolute values and as a percentage of the predicted values referred to European Respiratory Society (ERS) reference values [58]. FeNO was measured using an electrochemical analyzer (HypairFeNOMedisoftExp’air, Dinant, Belgium) according to ATS/ERS recommendations for online measurement of exhaled NO in adults [59]. Peripheral blood leukocyte counts were measured using an automated hematology analyzer.

**Table 1:** Demographic data, clinical characteristics and laboratory data of asthma patients and healthy controls

Characteristics	Asthma Patients	Healthy Controls
Age—years	50.7 ± 15	49.1 ± 15
Female sex (%)	71	77
BMI (Kg/m^2^)	27.17 ± 5	27.51 ± 4.2
Number of hospital admissions for asthma at any time before enrollment	0.5 ± 0.9	None
ACT score	14.5 ± 5.3	NA
Patients with concomitant nasal polyposis (%)	41.7	None
FEV_1_ (% of predicted value)	69.7 ± 4.2****	103 ± 11
F_ENO_ (ppb)	48.4 ± 23****	8.1 ± 3
Blood neutrophil count (cells/mm^3^)	4,160 ± 401***	3,371 ± 955
Blood eosinophil count (cells/mm^3^)	384 ± 18****	130 ± 78
Total IgE (IU/ml)	264.3 ± 70****	69.2 ± 107

NA: not applicable; *p < 0.05; **p < 0.01; ***p < 0.0005; ****p < 0.0001

**Table 2:** Demographic data, clinical characteristics and laboratory data of individual asthma patients

Subject	Age Years	Sex	FEV_1_ % predicted	FVC % predicted	FEV_1_/FVC	Atopy Yes/No	IgE IU/ml	Blood Eosinophils	Blood Neutrophils	ICS use Yes/No	ICS use μg daily FP equivalent	GCS use Yes/No	LABA use Yes/No	LTRA use Yes/No	LAMA use Yes/No
1	50	M	92	86	83.9	Yes	185	115	3210	No	0	No	No	No	No
2	47	F	64	78	76.4	Yes	290	140	3150	Yes	200	No	Yes	Yes	No
3	57	F	68	70	77,8	No	288	660	3990	Yes	320	No	Yes	No	No
4	21	F	68	74	79,9	Yes	138	330	1910	No	0	No	No	No	No
5	63	F	63	76	64.8	No	104	360	5010	Yes	320	Yes	Yes	No	No
6	65	F	36	49	73	No	144	610	9040	Yes	200	Yes	Yes	No	Yes
7	65	F	36	51	71	Yes	261	140	5090	Yes	320	No	Yes	Yes	No
8	65	F	66	77	67	Yes	83	180	3010	Yes	99	No	Yes	No	No
9	22	M	62	83	63.2	No	407	880	2660	No	320	No	Yes	No	No
10	45	F	85	104	65.7	Yes	377	870	3090	Yes	0	No	No	No	No
11	59	F	41	61	53.9	Yes	136	100	2420	Yes	200	No	Yes	Yes	Yes
12	44	M	109	105	83.6	Yes	116	580	3090	Yes	200	No	Yes	No	No
13	63	F	71	75	75	Yes	34	40	3820	Yes	320	No	Yes	No	No
14	57	F	89	88	79	Yes	709	360	5520	Yes	0	No	No	No	Yes
15	66	F	49	72	52.6	Yes	110	470	4880	Yes	320	No	Yes	Yes	No
16	47	F	81	77	84.8	Yes	150	620	4090	Yes	184	No	Yes	No	Yes
17	64	M	54	67	62.8	Yes	198	340	63.7	Yes	480	No	Yes	No	No
18	33	F	96	97	82.4	Yes	126	120	2530	Yes	320	No	Yes	No	No
19	61	F	72	71	80	Yes	1420	70	9100	Yes	320	No	Yes	No	Yes
20	43	M	111	97	90	Yes	93	130	3740	Yes	184	No	Yes	No	No
21	62	F	46	56	64.1	Yes	166	790	2680	Yes	184	Yes	Yes	No	No
22	32	M	65	78	68.6	Yes	654	350	4940	Yes	640	Yes	Yes	Yes	No
23	21	F	86	94	80.3	Yes	26	430	4760	No	0	No	No	No	No
24	65	M	63	77	63	Yes	99	740	3890	Yes	200	No	Yes	No	No
Mean ± SEM	50.7 ± 15	F 71%	69.7 ± 4.2	77.6 ± 15	72.6 ± 10	Yes: 20 (83%) No: 4 (17%)	264.3 ± 70	384 ± 18	4,160 ± 401	Yes: 20 (83%) No: 4 (17%)	222 ± 159	Yes: 4 (17%) No: 20 (83%)	Yes: 5 (21%) No: 19 (79%)	Yes: 5 (21%) No: 19 (79%)	Yes: 5 (21%) No: 19 (79%)

*FEV*_*1*_, Forced expiratory volume in the 1st second; *FVC*, Forced vital capacity; *ICS*, Inhaled glucocorticoids; *FP*, Fluticasone propionate; *GCS*, Oral glucocorticoids; *LABA*, Long-acting beta2 agonist; *LTRA*, Leukotriene-receptor antagonist; *LAMA*, Long-acting muscarinic agonist

### Neutrophil purification and culture

The study protocol, involving the use of human blood cells was approved by the Ethics Committee of the University of Naples Federico II (198/18), and written informed consent was obtained from blood donors according to the principles expressed in the Declaration of Helsinki. Leukocytes from peripheral blood of asthma patients and healthy controls were separated from erythrocytes by dextran sedimentation. Neutrophils were purified by Ficoll-Paque Histopaque®-1077 (Sigma-Aldrich, Milan, Italy) density gradient centrifugation (400 × g, 30 min, + 22 °C), followed by Percoll (Sigma-Aldrich, Milan, Italy) (65%) density gradient centrifugation (660 × *g*, 20 min, + 22 °C) as previously described [60]. Neutrophils were isolated from granulocytes (to reach > 99% purity) by positive elimination of all contaminating cells using the EasySep Neutrophil Enrichment Kit (StemCell Technologies, Vancouver, Canada) [61]. These cells were > 99% neutrophils as evaluated by flow cytometric analysis with the following antibodies: anti-CD3, anti-CD14, anti-CD15, anti-CD11b, anti-CD193 (Miltenyi Biotec, Bergisch Gladbach, Germany), anti-CD62L (L-selectin) (BD Biosciences, San Jose, CA, USA) and anti-CD66b (BioLegend, CA, USA). Samples were analyzed on the MACSQuant Analyzer 10 (Miltenyi Biotec, Bergisch Gladbach, Germany) and in the FlowJo software, v.10. Dead cells, doublets, debris and eosinophils were excluded from the analysis. Data were expressed as percentage of positive cells or median fluorescence intensity [62].

### Reactive oxygen species (ROS) production

Neutrophils (2 × 10^6^ cells/mL) were resuspended in the RPMI 1640 medium with 5% of fetal bovine serum (FBS) (Euroclone, Milan, Italy) and antibiotics at 37 °C and 5% CO_2_. The cells were incubated for 30 min after the addition of H_2_DCF-DA 10 µg/mL (Life Technologies, Milan Italy). H_2_DCF-DA is a fluorogenic dye that allows to determine hydroxyl peroxyl and other ROS activities within the cell [60]. Once diffused into the cell, H_2_DCF-DA is deacetylated by cellular esterases to a non-fluorescent molecule, which is oxidized by ROS into 2’,7’ dichlorofluorescein (DCF). DCF, a highly fluorescent molecule, is oxidized by fluorescent spectroscopy with maximum excitation and emission wavelengths of 492–495 and 517–527 nm, respectively. Neutrophils were washed in PBS and resuspended in RPMI 5% FBS in the presence or absence of different immunologic (LPS; 100 ng/ml) or fMLP; 1 µM) or non-immunologic stimuli (phorbol 12-myristate 13-acetate: PMA; 10 ng/ml) (Sigma-Aldrich, Milan, Italy) and immediately analyzed in a 96-well plate and placed in a EnSpire Multimode Plate Reader (Perkin Elmer, Waltham, MA, USA). DCF mean fluorescence intensity was measured at an excitation wavelength of 492–495 nm and emission at 517–527 nm. The ability of LPS and fMLP to induce cytoplasmic ROS-catalyzed oxidation of DCFH in neutrophils was measured as compared to negative control.

### Flow cytometry

Highly purified (> 99%) neutrophils were kept in RPMI with 10% of FCS for 30 min at + 37 °C, then were washed with PBS solution and stimulated with LPS (100 ng/ml), fMLP (1 µM) or PMA (10 ng/ml). Cells maintained in RPMI 1640 medium containing 5% of FBS (Euroclone, Milan, Italy) were the negative control (60 min, + 37 °C). 2 × 10^5^ cells were seeded in 96-well plate (Thermo Fisher Scientific, Waltham, MA, USA) and Zombie Violet dye (BioLegend, CA, USA) was added to evaluate cell viability (20 min, + 4 °C). Then, the cells were stained (20 min, + 4 °C) in PBS containing 1% FBS. The following antibodies were used: allophycocyanin (APC)-conjugated anti-CD66b (clone REA306, dilution 1:50, from Miltenyi Biotech, Bergisch Gladbach, Germany), peridinin chlorophyll protein (PerCP)-conjugated anti-CD11b (clone REA713, dilution 1:50, from Miltenyi Biotech, Bergisch Gladbach, Germany), fluorescein isothiocyanate (FITC)-conjugated anti-CD62L (clone 145/15, dilution 1:50, from Miltenyi Biotech, Bergisch Gladbach, Germany) and Phycoerythrin (PE)-conjugated anti-CD16 (clone REA423, dilution 1:50, from Miltenyi Biotech, Bergisch Gladbach, Germany). Cells were acquired by MACS Quant Analyzer 10 (Miltenyi Biotec, Bergisch Gladbach, Germany) and analyzed by FlowJo v.10 software. Doublets and debris (identified based on forward and side scatter properties), dead cells (identified with Zombie Violet Fixable Viability Kit; BioLegend) and eosinophils (identified based on the CCR3^+^ exclusion gate) were excluded from the analysis. A representative example of the complete gating strategy is illustrated in Supplementary Fig. 1. Data are expressed as a percentage of positive cells or median fluorescence intensity [62].

### Measurement of neutrophil-related mediators

Plasma levels of myeloperoxidase (MPO), matrix metalloproteinase 9 (MMP-9), CXCL8/IL-8, granulocyte–monocyte colony-stimulating factor (GM-CSF) and vascular endothelial growth factor (VEGF-A) of asthma patients and healthy controls were measured using ELISA kits according to the manufacturer’s instructions (DuoSet ELISA kit, R&D Systems, Minneapolis, MN, USA). A microplate reader (Tecan, Grodig, Austria, GmbH) was used to determine sample absorbance at 450 nm. The ELISA detection range was 62.5–4000 pg/ml (MPO), 31.2–2000 pg/ml (MMP-9), 31.2–2000 pg/ml (CXCL8/IL-8), 7.80–500 pg/ml (GM-CSF) and 31–2000 pg/ml (VEGF-A).

### Plasma NET detection

Plasma circulating free DNA (dsDNA), a marker for NET formation [63, 64], was measured using a Quant-iT™ PicoGreen dsDNA Assay Kit (Thermo Fisher Scientific, Waltham, MA, USA) as described in the manufacturer’s instructions. Concentration of Citrullinated Histone H3 (CitH3) in plasma samples of asthma patients and healthy controls was measured using ELISA kit developed by Cayman Chemicals (Ann Arbor, MI, USA) according to the manufacturer’s instructions. This assay used a specific monoclonal antibody for histone H3 citrullinated at residue R2, R8 and R17 (clone 11D3). A microplate reader (Tecan, Grodig, Austria, GmbH) was used to determine sample absorbance at 450 nm. The ELISA sensitivity is 0.15–10 ng/ml. The H3Cit measurement was set to 0 ng/ml when the absorbance of a patient sample was lower than the buffer blank. All measurements were executed in duplicates and results are expressed as ng/ml.

### Statistical analysis

Statistical analysis was performed by using Prism 7 (GraphPad software). Data are expressed as mean ± SEM of the indicated number of experiments. D'Agostino & Pearson normality test was used to test for normality of distribution, and statistical methods were chosen to fit non-normal distributions when appropriate. Values from groups were compared by Student’s t test or Mann–Whitney U test based on the parametric or nonparametric distribution of the continuous variables. Repeated measures one-way or two-way ANOVA was used were appropriated and described in the figure legends. Differences were assumed to be statistically significant when *p* < 0.05.

## Results

### Clinical characteristics of the study subjects

In this study, we enrolled 24 asthma patients and 22 healthy matched controls. Table 1 summarizes their clinical characteristics. The mean age of asthmatic patients was 50.7 ± 15 years and 71% were female. Asthma patients had 0.5 ± 0.9 hospital admissions for asthma exacerbation at any time before enrollment. The ACT score was 14.5 ± 5.3 and 41.7% of patients had concomitant nasal polyposis. The % of predicted value of FEV_1_ was significantly lower in asthmatics compared to healthy donors (69.7 ± 4.2% *vs*. 103 ± 11%; p < 0.0001). The median F_ENO_ was 48.4 ± 23 ppb. The absolute neutrophils count in asthma patients was increased than normal subjects (4,160 ± 401 *vs*. 3,371 ± 955; p < 0.0005). The absolute eosinophil count was significantly higher in asthmatics compared to healthy subjects (384 ± 18 *vs.* 130 ± 78; *p* < 0.0001). Serum IgE levels were 264.3 ± 70 IU/ml in asthmatic patients and 69.2 ± 107 IU/ml in control subjects (*p* < 0.0001). Demographics, clinical characteristics and laboratory data of individual asthma patients are presented in Table 2. Twenty patients (83%) were atopic; 20 patients (83%) were treated with inhaled glucocorticoids (ICS) and four patients (17%) were treated with oral glucocorticoids (OCS). None of the patients has been or was treated with allergen-specific immunotherapy or monoclonal antibodies anti-IgE, anti-IL-5/IL-5Rα or anti-IL-4Rα.

### ROS production by human neutrophils

**Fig. 1:**
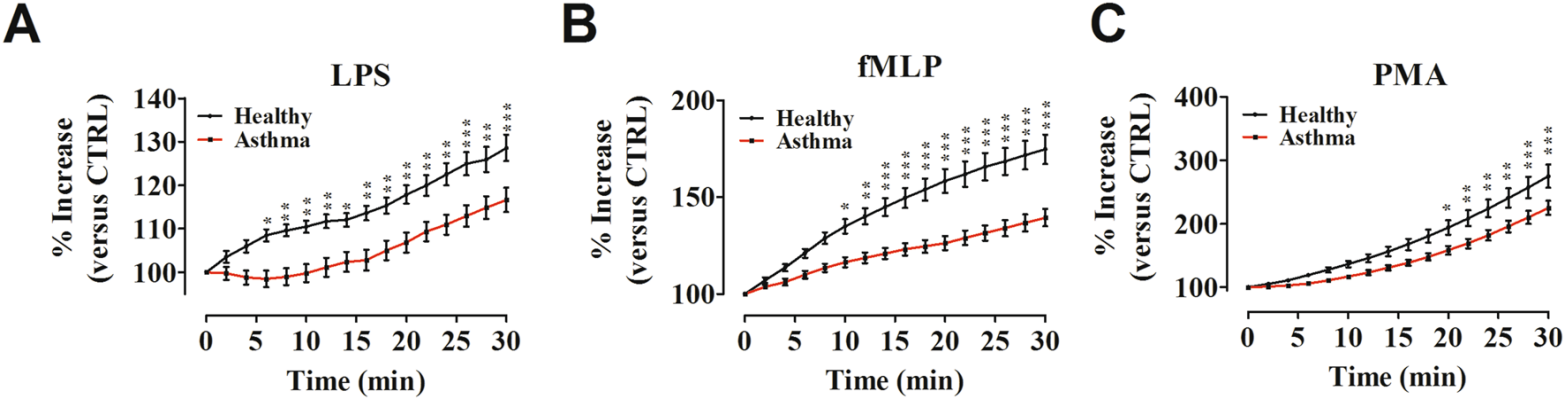
Neutrophils purified from peripheral blood of asthma patients (red lines) and healthy controls (black lines) were incubated (30 min, 37 °C) with 2’,7’-dichlorodihydrofluorescein diacetate (H_2_DCFDA, 10 µM), washed and then stimulated with LPS (100 ng/mL) (**A**), fMLP (1 µM) (**B**) or PMA (10 ng/mL) (**C**). Immediately after stimulation, PMNs were analyzed in a multimode microplate reader (EnSpire Multimode Plate reader, PerkinElmer) and DCF fluorescence was measured for 30 min at 2 min intervals. The results were expressed as percentage increase versus time 0 (mean ± SEM); *p < 0.05; **p < 0.01; ***p < 0.005. Two-way ANOVA and Bonferroni post-test

ROS are key signaling molecules that play important roles in the progression of several inflammatory disorders [65], including certain forms of asthma [49]. Highly purified neutrophils (> 90%) from asthma patients and healthy controls were activated by exposure to LPS, which activates toll-like receptor 4 (TLR4) [66] and to fLMP, which activates formyl peptide receptors [27]. Cells were also activated by PMA, a protein kinase C (PKC) agonist [25]. To test whether neutrophil activation leads to ROS production in asthma patients, we performed a 2′,7′-dichlorodihydrofluorescein diacetate (H_2_DCF-DA) ROS detection assay. Figure 1 illustrates the kinetics (2 to 30 min) of the production of ROS induced by LPS (Fig. 1A), fMLP (Fig. 1B) and PMA (Fig. 1C). ROS production by PMNs was significantly increased by all the inflammatory stimuli in asthma patients as well as in healthy controls. ROS production induced by LPS progressively increased from 2 to 30 min in normal PMNs. The production of ROS from PMNs isolated from asthma patients had a lag period of approximately 15 min and then progressively increased. PMNs purified from asthma patients increased ROS production but to a lower extent compared to neutrophils obtained from healthy donors. Figure 1A shows that the difference in LPS-induced ROS production in PMNs from asthma patients and healthy controls was evident from 6 min onward after stimulation. The shape of kinetics of fMLP-induced ROS production from controls was different from that caused by LPS. There was a rapid increase that tended to plateau after 30 min of incubation. fMLP-induced ROS production was stronger compared to LPS-induced ROS production but the difference between patients and controls was evident starting from 10 min onward after stimulation (Fig. 1B). PMA, a non-immunologic PKC activator, induced ROS production from neutrophils. Figure 1C shows that also the kinetics of PMA-induced ROS production from asthma patients and from healthy controls were different from those induced by LPS and fMLP. There was a slow increase that did not plateau after 30 min of incubation. ROS production was significantly higher in healthy subjects compared to asthmatics from 20 min onward after PMA stimulation. No difference in ROS production was evident between patients and controls when medium alone was used as negative control (data not shown). Collectively, these findings suggested that ROS production from asthma patient PMNs was lower compared with healthy controls when cells were activated by immunologic and non-immunologic stimuli.

### Neutrophil activation in asthma patients

**Fig. 2:**
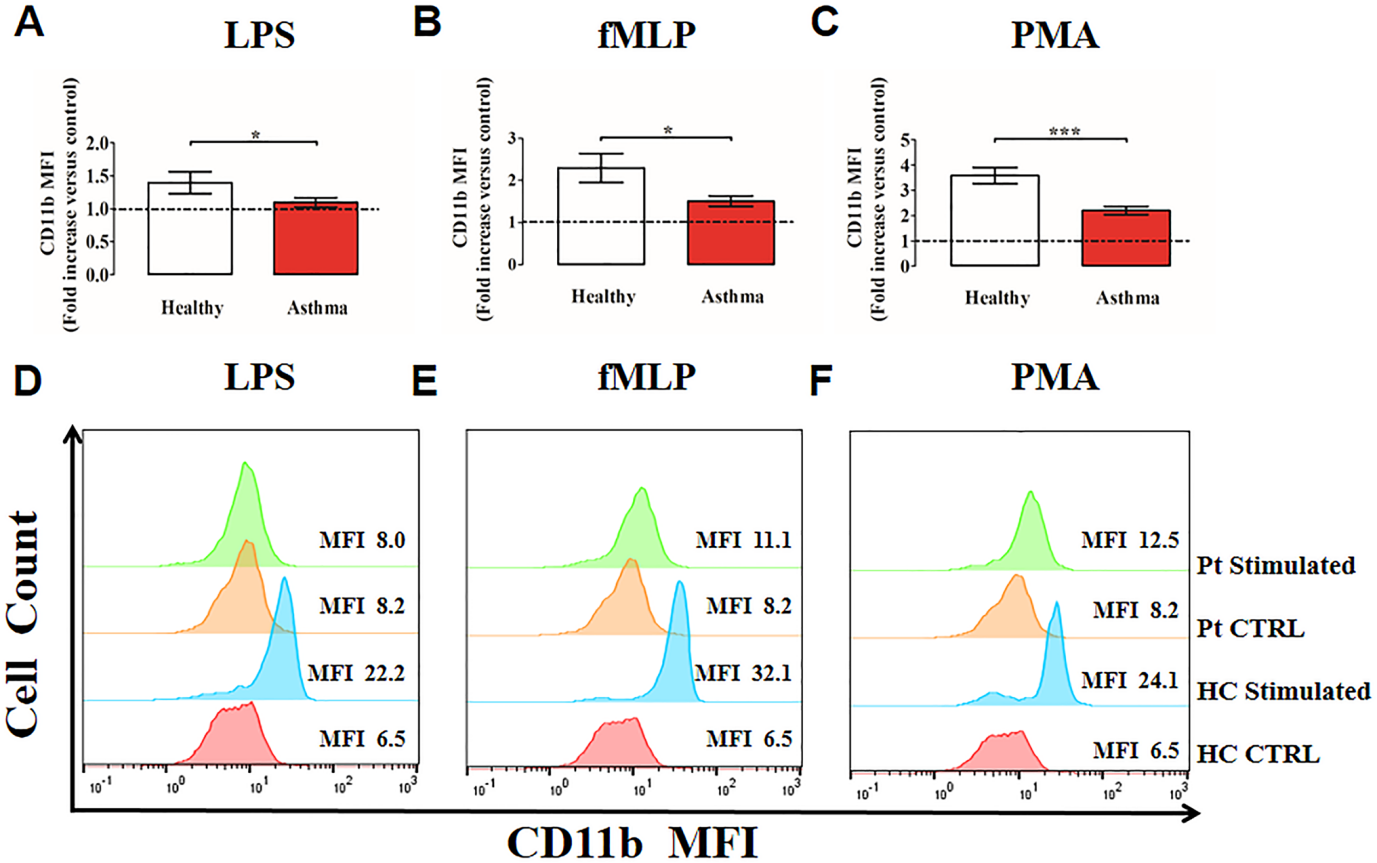
Neutrophils purified from peripheral blood of asthma patients (red bars) and healthy controls (white bars) were stimulated with LPS (100 ng/mL) (**A**), fMLP (1 µM) (**B**) or PMA (10 ng/mL) (**C**) for 60 min at 37 °C and then stained for the neutrophil activation marker CD11b and subjected to cytofluorimetric analysis. Mean fluorescence intensity (MFI) was calculated and normalized for non-stimulated cells (control). The control was set as 1 (dashed line). Results were expressed as fold increase *versus* control (mean ± SEM); *p < 0.05; ***p < 0.005. Student’s t test or Mann–Whitney U test according to the parametric or nonparametric distribution of the variables. **D–F.** Representative histograms illustrating MFI for CD11b expression on peripheral blood neutrophils from healthy controls (HC) and asthma patients (Pt), unstimulated (CTRL) or stimulated with LPS (**D**), fMLP **(E)** or PMA **(F).** MFI = Mean fluorescence intensity. CTRL = Control cells; HC = Healthy controls. Pt = Patients

To determine the activation status of human PMNs isolated from peripheral blood of asthma patients, we determined CD11b, CD16 and CD62L (L-selectin) expression on PMNs by flow cytometry [67, 68]. PMNs stimulated with PMA (as a positive control), fMLP, LPS or control medium, were stained with antibodies against CD11b, CD16 and CD62L and evaluated by flow cytometry. [69]. Under basal conditions, neutrophils from controls and asthmatics showed minimal expression of CD11b, which rapidly increased after incubation with all inflammatory stimuli tested. PMNs purified from asthma patients increased CD11b expression to a lower extent compared with healthy controls under LPS (Fig. 2A), fMLP (Fig. 2B) and PMA (Fig. 2C) stimulation. Figure 2D–F shows representative histograms illustrating CD11b expression on peripheral blood neutrophils from healthy controls (HC) and asthma patients (Pt), non-stimulated (CTRL) or stimulated with LPS (D), fMLP (E) and PMA (F)**.**

**Fig. 3:**
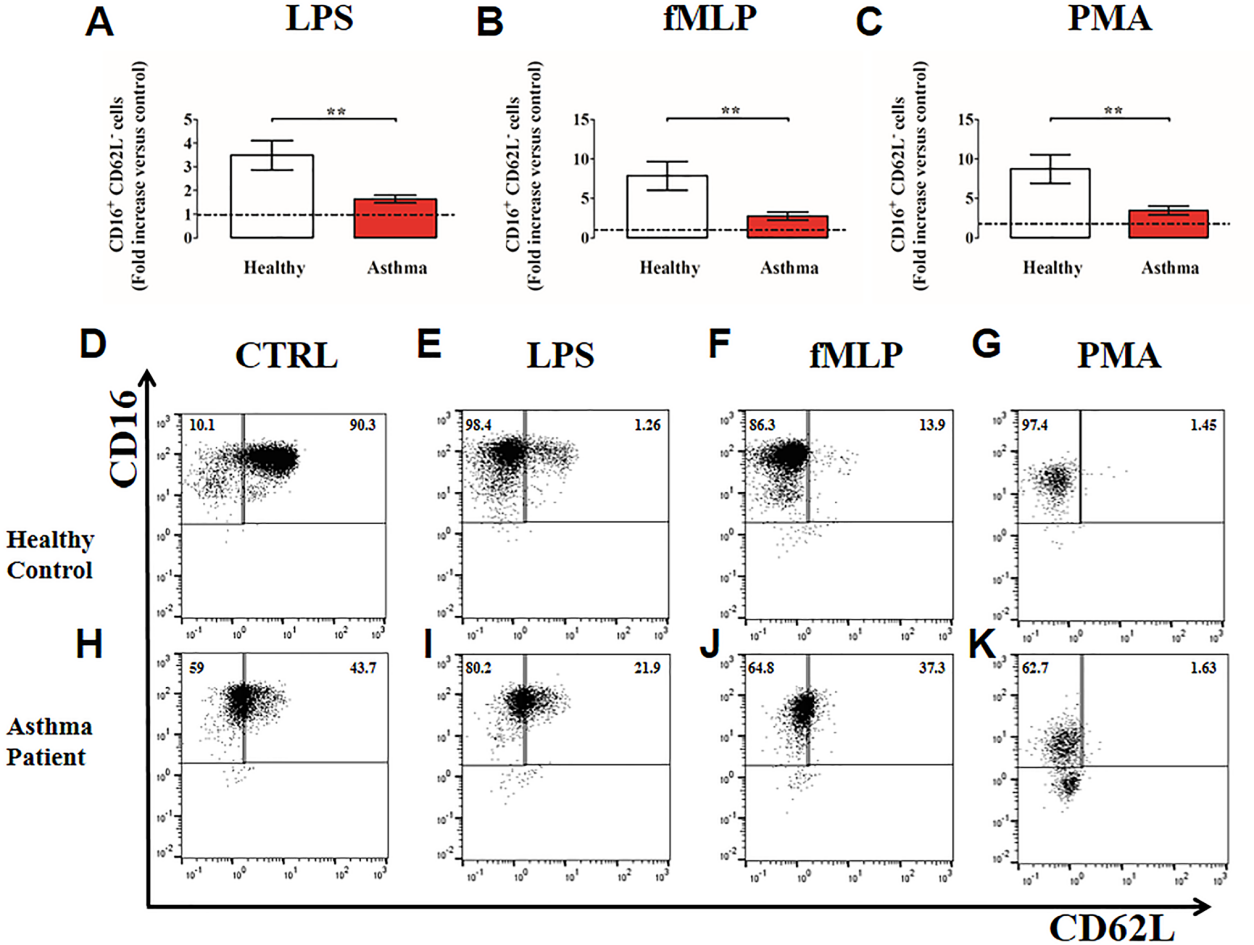
Neutrophils purified from peripheral blood of asthma patients (red bars) and healthy controls (white bars) were stimulated with LPS (100 ng/mL) (**A**), fMLP (1 µM) (**B**) or PMA (10 ng/mL) (**C**) or control medium for 60 min at 37 °C and then stained for neutrophil activation markers CD16 and CD62L and subjected to cytofluorimetric analysis. Percentages of CD16^+^CD62L^−^ cells were calculated and normalized for controls. The control was set as 1 (dashed line). The results were expressed as fold increase *versus* control (mean ± SEM); ** *p* < 0.01. Student’s t test or Mann–Whitney U test according to the parametric or nonparametric distribution of the variables. **D–K**: Representative flow cytometric panels with respect to the gating strategy and cell counts for healthy controls **(D, E, F,G**) and asthma patients (**H, I, J, K**), stimulated with control medium (**D, H**), LPS (**E, I**), fMLP **(F, J)** or PMA **(G, K)**

Under resting conditions, neutrophils expressed CD62L, which rapidly decreased (i.e., shedding) after stimulation with the three examined stimuli. Indeed, the CD16^bright^/CD62L^dim^ cells consist mainly of neutrophils containing hyper-segmented nuclei that show a more activated state [70]. Interestingly, under LPS, fMLP or PMA stimulation, PMNs purified from asthma patients underwent CD62L shedding, but to a lesser extent compared to healthy donors (Fig. 3A–C). Figure 3D–K shows representative flow cytometric panels with respect to the gating strategy and CD16^+^CD62L^−^ cell counts for healthy controls (D–G) and asthma patients (H–K), stimulated with LPS (E, I), fMLP (F, J) and PMA (G, K) or control medium (D, H). Collectively, these data indicate that when challenged with inflammatory stimuli, peripheral blood PMNs from asthmatics displayed a reduced activation status (CD11b up-regulation and CD62L shedding) compared to PMNs purified from controls.

### Plasma levels of neutrophil-derived mediators

**Fig. 4:**
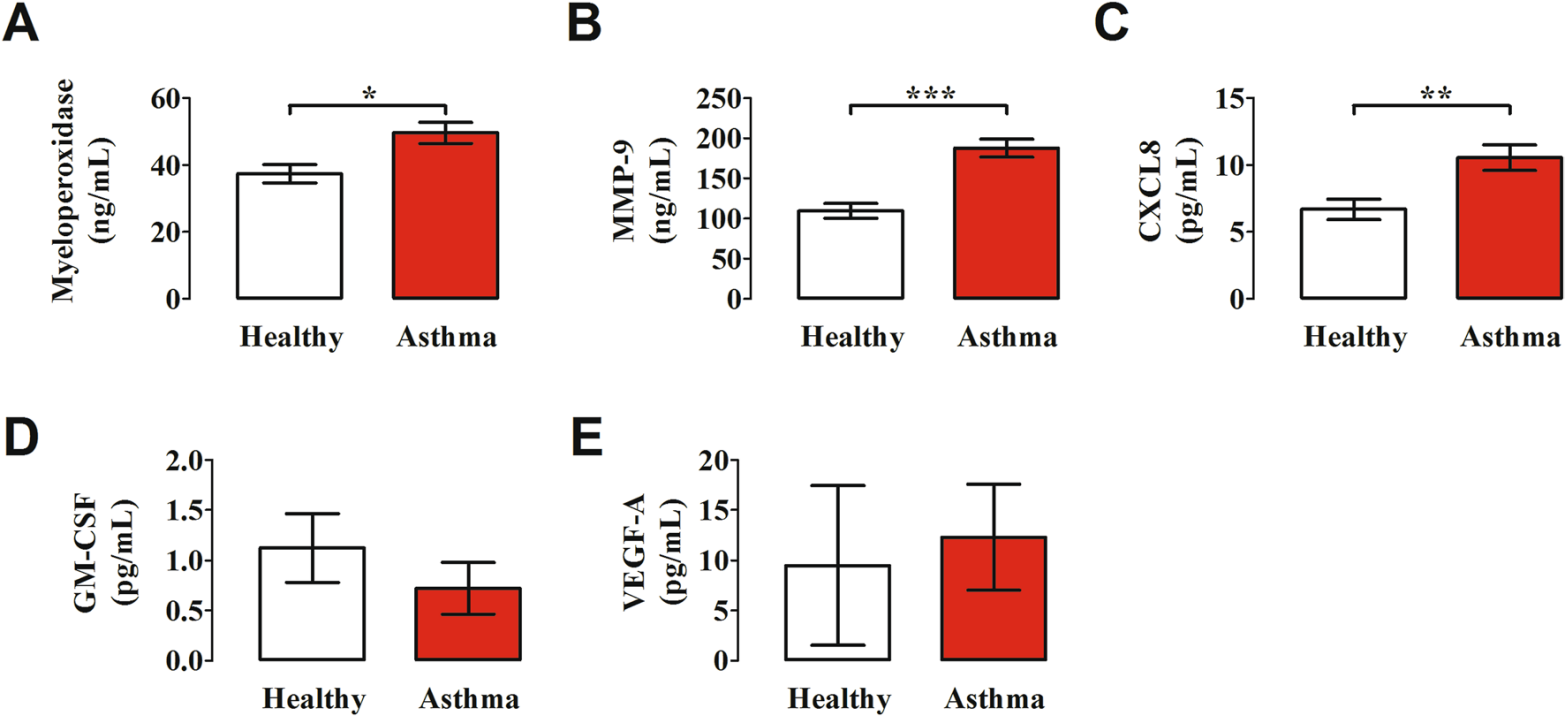
Plasma concentrations of myeloperoxidase (**A**), MMP-9 (**B**), CXCL8 (**C**), GM-CSF (**D**) and VEGF-A (**E**) in asthma patients (red bars) and healthy controls (white bars) were measured by ELISA. The results were expressed as mean ± SEM. *p < 0.05; **p < 0.01; *** *p* < 0.005. Student’s t test or Mann–Whitney U test according to the parametric or nonparametric distribution of the variables

Human neutrophils contain and release of a variety of proinflammatory mediators [28, 71]. Myeloperoxidase (MPO), contained in neutrophil primary/azurophilic granules, plays a pivotal role in ROS production [72, 73] and NET formation [74, 75]. Matrix metalloproteinase-9 (MMP-9) plays a key role in the development of airway inflammation [76–79] and facilitates angiogenesis in asthma [80]. Circulating [81] and BAL levels of CXCL8 [82] are increased in asthmatics. GM-CSF is considered a key orchestrator of neutrophil involvement in chronic airway inflammation [83, 84]. VEGF-A, the most important angiogenic factor [85–87], is produced by activated human neutrophils [66, 88, 89]. Figure 4 shows that the plasma levels of MPO, MMP-9 and CXCL8 were significantly increased in asthmatics compared to healthy donors. By contrast, the plasma concentrations of GM-CSF and VEGF-A did not differ between the two groups of subjects examined. Collectively, these results suggest that neutrophils from asthmatics are activated in vivo to release neutrophil-derived mediators.

### Plasma levels of NET components

**Fig. 5:**
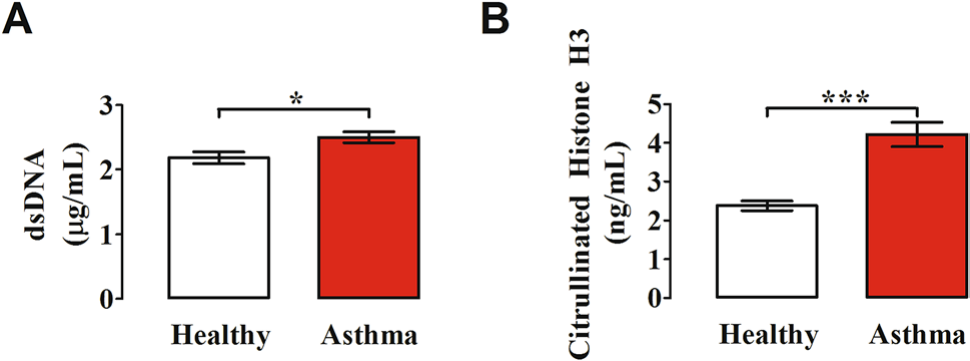
Plasma concentrations of circulating free DNA (cfDNA) (**A**) and citrullinated histone H3 (CitH3) (**B**) in asthma patients (red bars) and healthy controls (white bars) were measured by Quant-iT™ PicoGreen™ dsDNA Assay Kit (Thermo Fisher) and Citrullinated Histone H3 (clone 11D3) ELISA kit (Cayman), respectively. Results were expressed as mean ± SEM; *p < 0.05; ***p < 0.005. Student’s t test or Mann–Whitney U test according to the parametric or nonparametric distribution of the variables

**Fig. 6:**
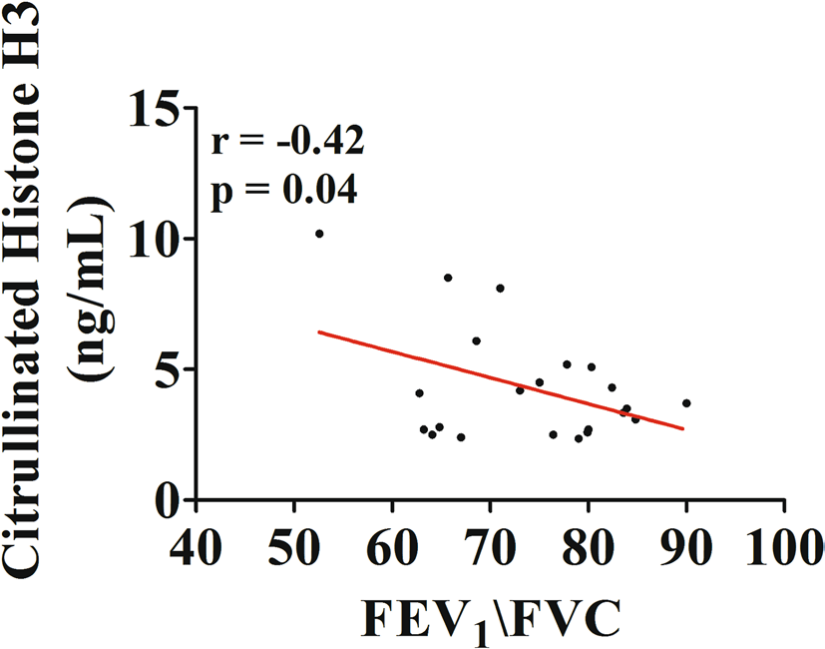
Correlation between plasma concentrations of Citrullinated Histone H3 (CitH3 and FEV_1_/FVC (%) in asthma patients. Spearman correlation coefficient with *r* = − 0.42; *p < 0.05

Human neutrophils activated by a variety of immunologic and non-immunologic stimuli release NETs [74, 75, 90, 91]. We evaluated the plasma levels of different components of NETs such as circulating free DNA [42] [92] and citrullinated histone H3 (CitH3), a more specific marker for NET formation [93]. Figure 5 shows that circulating levels of dsDNA were higher in asthma patients compared to healthy donors (Fig. 5A). Even more marked were the increased plasma levels of CitH3 in asthmatics compared to controls (Fig. 5B). Interestingly, the circulating concentrations of CitH3 were inversely correlated with a parameter FEV_1_/FVC of lung function in asthmatics (Fig. 6).

## Discussion

Highly purified neutrophils isolated from peripheral blood of asthma patients and healthy donors produced ROS in response to two agonists (LPS and fMLP) that activate specific receptors (TLR4 and FPR, respectively) expressed on the cytoplasmic membrane of these cells [24, 66]. The production of ROS was reduced in PMN from asthmatics compared to healthy donors. Similarly, the mobilization of two surface markers of neutrophils, CD11b and CD62L, induced by LPS and fMLP, were more marked in cells from healthy donors compared to asthmatics. Collectively, these results are compatible with the hypothesis that circulating neutrophils from asthma patients are in vivo activated and desensitized. Circulating levels of neutrophil-derived mediators, MPO, MMP-9 and CXCL8, were increased in asthmatics compared to controls. Plasma levels of two NET components, dsDNA and CitH3, a specific measure for NET formation, were increased in asthmatics and inversely correlated with a parameter of lung function in asthmatics.

Human neutrophils are a prominent source of ROS, being capable of producing significant quantities of superoxide anion (O_2_^−^), hydrogen peroxide (H_2_O_2_) and hypochlorous acid (HOCl) [23, 94]. These species are indispensable component of host defense against microorganisms, but are also capable of tissue damage in different murine models of asthma [52, 95]. A limited study performed in 8 asthmatics and 7 controls reported that fMLP- and PMA-induced O_2_^−^ production by circulating neutrophils was increased in asthma patients [96]. A similar study in 11 asthmatics confirmed that fMLP- and PMA-induced production of O_2_^−^ by neutrophils was greater in asthma patients [97]. In asthmatics, the production of O_2_^−^ inversely correlated with FEV_1_. More recently, it has been shown that peripheral blood neutrophils from asthmatics activated by fMLP and LPS induced an increase in ROS production [49]. Our results confirm and extend the previous observations by showing that two receptor-mediated stimuli (i.e., fMLP and LPS) and PMA, a direct activator of neutrophil PKC, caused a time-dependent production of ROS from highly purified neutrophil isolated from asthmatics and normal subjects. However, differently from previous studies [96, 97], the production of ROS in PMNs from asthmatics induced by the three stimuli was decreased compared to normal donors. Different experimental techniques and study design might explain, at least in part, these apparently different results. For instance, previous studies evaluated the O_2_^−^ generation by human neutrophils [96, 97], whereas we and others [49] have measured the intracellular production of ROS in highly purified neutrophils obtained from larger cohorts of donors. Alternatively, there is the possibility that the pharmacologic treatment of asthma patients may have altered the production of ROS from neutrophils. For instance, ICS and/or oral glucocorticoid treatment in the vast majority (> 80%) of our patients may have reduced ROS production from neutrophils. We would like to suggest that circulating neutrophils from asthmatics are in vivo chronically stimulated by various stimuli which render these cells hyporesponsive to in vitro stimulation.

To support this hypothesis, we evaluated the changes of the neutrophil expression of CD11b and CD62L induced by LPS, fMLP and PMA in asthmatics and controls. Under basal conditions, there is a minimal surface expression of CD11b [60]. All three stimuli activated neutrophils inducing the over-expression of CD11b, which was more marked in healthy controls compared to asthma patients. CD62L is normally expressed by the majority of resting neutrophils and is down-regulated following cell activation. The three stimuli induced a more marked CD62L shedding in neutrophils from healthy donors compared to asthmatics. Collectively, these results support the hypothesis that neutrophils from asthmatics are presumably in vivo desensitized and are less responsive to in vitro activation by various stimuli.

Our experiments were performed on normal density neutrophils (NDNs) isolated by Ficoll-Paque Histopaque® density gradient centrifugation. Several groups of investigators have identified and characterized a subset of human peripheral blood PMNs named low-density neutrophils (LDNs) [70, 98–101]. It has been suggested that LDNs represent a subset of activated neutrophils compared to NDNs [102]. Interestingly, fMLP selectively up-regulated CD11b and down-regulated CD62L expression in LDNs. Based on these findings, we cannot exclude the possibility that some of the differences that we found in the LPS/fMLP/PMA-induced expression of CD11b and/or CD62L in neutrophils could be due to changes in the percentage of NDNs and LDNs in asthmatics.

Neutrophils are a rich source of preformed granular constituents which can be rapidly released [23]. MPO is a cationic enzyme localized in primary azurophilic granules, which catalyzes ROS production [73] and contributes to NET formation [24, 103]. In this study, we found that plasma levels of MPO are increased in asthmatics compared to healthy controls. A recent study demonstrated that sputum concentrations of MPO are increased in patients with asthma and correlated with a NET-derived biomarker [45].

MMP-9, present in tertiary granules of neutrophils, was found in BAL of asthmatics [79] and it was correlated to the absolute neutrophil count [104], MMP-9^−/−^ mice showed reduced immune cell infiltration and bronchial hyperresponsiveness compared to wild type mice in a murine model of asthma [76]. MMP-9 was increased in BAL and in sputum of asthmatics after allergen challenge [77, 78]. Moreover, neutrophil-derived MMP-9 was increased in severe asthma [104]. In this study, we found that plasma concentrations of MMP-9 are increased in asthma patients compared to controls.

Human neutrophils contain and release CXCL8 [105]. Asthmatic individuals have higher circulating [81, 106–108], sputum [109] and BAL fluid [82] CXCL8 concentrations compared to healthy controls. Our results indicate that plasma levels of CXCL8 are increased in asthmatics compared to healthy controls. Collectively, these results showing that three different neutrophil-derived mediators are increased in asthma patients suggest that PMNs are in vivo activated.

GM-CSF is a pleiotropic cytokine that plays a role in the differentiation, activation and survival of several immune cells, including neutrophils [110]. GM-CSF levels are higher in sputum, BAL fluid and bronchial tissue in patients with asthma [111–113]. Recent studies indicate that GM-CSF plays a central role in experimental models of asthma [83, 84]. In our cohort of asthmatics, circulating levels of GM-CSF are comparable to controls. It is conceivable that larger patient cohorts may reveal significant differences. Alternatively, it is possible to hypothesize that GM-CSF plays a role in asthma as a local (e.g., pulmonary) rather than a circulating cytokine.

Angiogenesis, the formation of new blood vessels, is a complex process modulated by a plethora of stimulatory and inhibitory factors [85, 86]. There is some evidence the angiogenic switch in asthmatic airways involves the production of direct (e.g., VEGFs, angiopoietins) and indirect angiogenic factors (e.g., MMP-9, cysteinyl leukotrienes) [86, 114, 115]. Interestingly, the plasma concentrations of VEGF-A, which is also released by activated human PMNs [88, 89], were similar in asthmatic and controls. Our finding that circulating levels of VEGF-A are not altered in asthma patients compared to healthy donors is rather intriguing. In fact, several immune cells involved in the pathogenesis of asthma such as mast cells [116, 117], macrophages [118], basophils [119], eosinophils [120] and neutrophils [66, 88, 89] are a major source of VEGF-A. Perhaps, the local production of angiogenic factors is more important than circulating levels of these factors in asthma. An alternative hypothesis to explain our results could arise from the observation that ROS up-regulate VEGF-A expression [121]. Therefore, the reduced production of ROS from neutrophils of asthmatics could explain, at least in part, the normal plasma levels of VEGF-A in these patients.

NETs were originally described as a protective mechanism by which neutrophils exerted an antibacterial role [25, 74]. During the last years, this initial interpretation has significantly evolved by showing that NETs can exert proinflammatory [24], as well as anti-inflammatory effects [122]. Moreover, human neutrophils can release lytic [25, 123] and non-lytic NETs [60, 91, 124]. There is growing evidence that NETs play multiple roles in several inflammatory diseases [37, 38, 125], cancer [24, 60, 92] and allergic disorders [42, 43, 45, 47, 126, 127]. NETs were first described in bronchial biopsy specimens from four atopic asthmatics expressing a high number of neutrophils [47]. dsDNA, a putative marker of NET formation, was found in induced sputum of asthmatics [126]. Johnston and collaborators demonstrated that nasal dsDNA levels were increased in nasal lavage after rhinovirus infection in asthmatics [42]. A collaborative study on severe asthma found that BAL dsDNA was increased in patients with neutrophilia [127]. A similar study reported that increased dsDNA in sputum from patients with severe asthma reflected local neutrophil activation [45]. Interestingly, a positive linear correlation was found between dsDNA and MPO concentrations. The latter observation is relevant because MPO is a key component of NET formation [29].

Different analytical methods (e.g., dsDNA, MPO-DNA, CitH3) for the measurement of NET markers in asthma were used by several groups [42, 45, 126, 128–130]. The measurement of dsDNA has been widely used as a NET marker [42, 45, 126]. However, the quantitative analysis of circulating DNA does not necessarily reflect the extent of NET formation in vivo. In fact, DNA complexes may result from any cell death associated with neutrophilic inflammation [128]. MPO-DNA has been proposed as a circulating NET marker of severe asthma [130]. However, the ELISA detection of MPO-DNA complexes in human plasma is error prone and yields limited information of NETs formed in vivo [129]. For the above reasons, we have used two different analytical methods (i.e., dsDNA and CitH3). Our results demonstrate that plasma concentrations of two putative markers of NETs, dsDNA and CitH3, are increased in asthma patients compared to healthy controls. In particular, we validated our findings by measuring plasma levels of CitH3 which is considered a more reliable biomarker of NET formation [24]. Collectively, these results indicate that increased circulating levels of two markers of NET formation are associated with bronchial asthma.

Putative markers of NETs have been detected in different sites/biological fluids of asthmatics. DNA presence was initially described in bronchial biopsies of adult asthmatics [47]. dsDNA have been found in sputum [45, 126], nasal lavages [42] and BAL of asthma patients [127, 130]. Granger et al. recently investigated the presence of putative NET markers in serum of asthma patients [130]. These authors examined MPO-DNA complexes by ELISA which is error prone and yields limited information of NETs in vivo [129]. Our study is, to the best of our knowledge, the only one in which two different analytical methods (i.e., dsDNA and CitH3) are used to evaluate plasma NETs in asthmatic patients. It is evident that the ease of access to plasma samples compared to BAL or induced sputum makes our results of potential clinical applicability.

Our findings might have additional translational relevance for a number of possible reasons. First, there is the possibility that measurement of circulation NET-derived products (i.e., CitH3) could represent an easily measurable plasma biomarker of asthma severity. In this context, we have found an inverse correlation between circulating levels of CitH3 and FEV_1_/FVC in asthmatics. Second, several inhibitors of NET formation and molecules that degrade NETs are under active investigation for the treatment of inflammatory disorders and cancer [24]. If further studies will demonstrate the pathogenic role of NETs in asthma, it is conceivable that some of the various strategies that modulate NET formation [24] could be envisioned for the treatment of allergic disorders.

At this point, the question obviously arises: what could be the stimuli that induce the in vivo formation of NETs in asthmatics? A plethora of immunologic and non-immunologic stimuli can induce NET formation in vivo. Among these, LPS [60, 75, 131, 132], IL-17 [133], IL-33 [134, 135], CXCL8 [60, 136–138], C3a/C5a [75, 139, 140] and IL-1β [45, 141], which have been implicated in various aspects of asthma [42, 45], are inducers of NET formation in neutrophils. Moreover, several bacterial products (i.e., fMLP) [92, 139, 142], rhinovirus and influenza virus can induce NET formation [42, 143]. Further studies are necessary to identify in vivo inducers of NET formation in asthmatics.

T2-high asthma is characterized by type 2 inflammation involving Th2 lymphocytes, ILC2s, mast cells, basophils, eosinophils, type 2 cytokines and IgE [8–10]. On the other hand, T2-low asthma is less characterized and might involve Th1 and/or Th17 cells, ILC1s and ILC3s [9, 15]. There is increasing evidence that this is a rather simplistic and procrustean classification. Our results emphasize a possible role of NETs and neutrophil-derived mediators also in allergic asthmatics. These findings could suggest that neutrophils could play a pathogenic role not only in T2-low but also in a percentage of T2-high asthmatics.

This study has several limitations that should be pointed out. The sample size of the asthma patient and healthy control cohorts investigated in the present study is limited. Further studies on larger cohorts of healthy donors and patients could highlight the significance of NET formation in these patients. Although our results indicate that increased CitH3 plasma levels were inversely correlated to reduced lung function, at this stage, it would have been inappropriate to define CitH3 as a biomarker of asthma severity. We anticipate that it would be necessary to examine at least one order of magnitude larger cohorts. Asthma is a heterogeneous group of inflammatory disorders characterized by complex pathology, distinct subtypes and highly variable clinical courses [144–147]. The patients included in this study were adults with a history of allergist-diagnosed asthma of different severity (mild, moderate, severe). No correlation was found between the plasma CitH3 concentrations and FeNO, serum IgE and peripheral blood eosinophils or neutrophils. The limited sample size of patients does not allow to correlate the increased CitH3 levels to T2-low or T2-high asthma. The contribution of NETs and the clinical relevance of CitH3 measurement as a biomarker in different forms of asthma remain to be investigated in larger cohorts of patients. Finally, two forms of NET formation have been identified: suicidal NETosis [25, 123] and vital NET release [60, 91, 124]. Further in vitro and in vivo studies should investigate which of these two distinct forms of NET formation are involved in different phenotypes of asthma.

In conclusion, our study indicates that circulating neutrophils from asthmatics released ROS when activated by immunologic and non-immunologic stimuli. Plasma levels of several biomarkers of neutrophils activation such as MPO, MMP-9 and CXCL8 were increased in asthma patients compared to healthy subjects. Circulating levels of two NET-derived products such as dsDNA and CitH3 were increased in asthmatics compared to controls and were inversely correlated to FEV_1_/FVC decrease. Together, these findings indicate that neutrophils and their products might have an active role in the pathophysiology of asthma.

## Supplementary Information

Below is the link to the electronic supplementary material.

Supplementary file1 (DOCX 1700 kb)

## Data Availability

All data generated or analyzed during this study are included in this published article.
