# Supplementary material for: Neutrophil extracellular traps and neutrophil-derived mediators as possible biomarkers in bronchial asthma
Source: Clin Exp Med. 2021 Aug 3;22(2):285–300. doi: 10.1007/s10238-021-00750-8 (PMC9110438; doi:10.1007/s10238-021-00750-8)

**Figure S1.** Representative flow cytometric panels with respect to the complete gating strategy for healthy controls and asthma patients. PMNs were purified from peripheral blood and stained with the monoclonal antibodies as indicated. Flow cytometry plots were gated on live single cells and show forward (FSC) and side scatter (SSC) of EasySep-purified untouched neutrophils (**A, B**). Since Vioblue-positive cells included both dead cells and CCR3^+^ cells (eosinophils), both cells were excluded based on a negative gate (**C**). Neutrophils were further identified as CD66b^+^CD11b^+^ cells (**D**) and analyzed for their expression of the activation markers CD16 and CD62L (**E**).


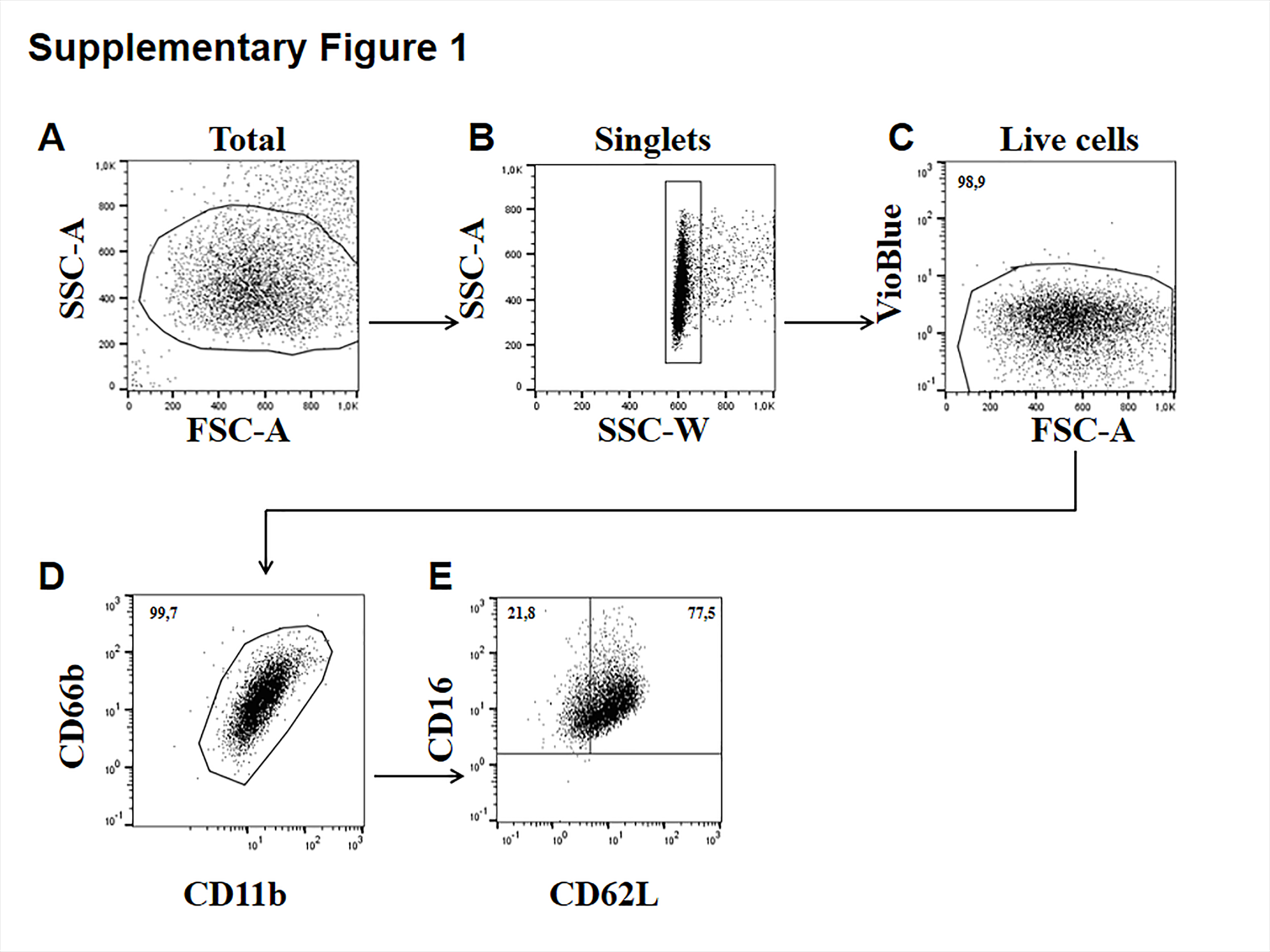

Supplement: Supplementary file 1 — Supplementary file1 (DOCX 1700 kb) [file 10238_2021_750_MOESM1_ESM.docx]
